# HDAC4 represses ER stress induced chondrocyte apoptosis by inhibiting ATF4 and attenuates cartilage degeneration in an osteoarthritis rat model

**DOI:** 10.1186/s12891-024-07578-9

**Published:** 2024-06-15

**Authors:** Xiaodong Gu, Fei Li, Xianda Che, Xiaochun Wei, Pengcui Li

**Affiliations:** 1 Department of Orthopaedics, Shanxi Bethune Hospital, Shanxi Academy of Medical Sciences,Third Hospital of Shanxi Medical University, Tongji Shanxi Hospital, Taiyuan, Shanxi Province 030032 People’s Republic of China; 2 Department of Orthopaedics, The Second Hospital of Shanxi Medical University, Taiyuan, Shanxi Province 030001 People’s Republic of China; 3 Shanxi Key Laboratory of Bone and Soft Tissue Injury Repair, Taiyuan, Shanxi Province 030001 People’s Republic of China

**Keywords:** Histone deacetylase 4, Activating transcription factor 4, Endoplasmic reticulum stress, Chondrocyte apoptosis, Osteoarthritis

## Abstract

**Background:**

The present study evaluated whether the lack of histone deacetylase 4 (HDAC4) increases endoplasmic reticulum stress-induced chondrocyte apoptosis by releasing activating transcription factor 4 (ATF4) in human osteoarthritis (OA) cartilage degeneration.

**Methods:**

Articular cartilage from the tibial plateau was obtained from patients with OA during total knee replacement. Cartilage extracted from severely damaged regions was classified as degraded cartilage, and cartilage extracted from a relatively smooth region was classified as preserved cartilage. Terminal deoxynucleotidyl transferase dUTP nick end labeling staining was used to detect chondrocyte apoptosis. HDAC4, ATF4, and C/EBP homologous protein (CHOP) expression levels were measured using immunohistochemistry staining and real-time quantitative PCR. Chondrocytes were transfected with HDAC4 or HDAC4 siRNA for 24 h and stimulated with 300 µM H_2_O_2_ for 12 h. The chondrocyte apoptosis was measured using flow cytometry. ATF4, CHOP, and caspase 12 expression levels were measured using real-time quantitative PCR and western blotting. Male Sprague-Dawley rats (*n* = 15) were randomly divided into three groups and transduced with different vectors: ACLT + Ad-GFP, ACLT + Ad-HDAC4-GFP, and sham + Ad-GFP. All rats received intra-articular injections 48 h after the operation and every three weeks thereafter. Cartilage damage was assessed using Safranin O staining and quantified using the Osteoarthritis Research Society International score. ATF4, CHOP, and collagen II expression were detected using immunohistochemistry, and chondrocyte apoptosis was detected using terminal deoxynucleotidyl transferase dUTP nick end labeling staining.

**Results:**

The chondrocyte apoptosis was higher in degraded cartilage than in preserved cartilage. HDAC4 expression was lower in degraded cartilage than in preserved cartilage. ATF4 and CHOP expression was increased in degraded cartilage. Upregulation of HDAC4 in chondrocytes decreased the expression of ATF4, while the expression of ATF4 was increased after downregulation of HDAC4. Upregulation of HDAC4 decreased the chondrocyte apoptosis under endoplasmic reticulum stress, and chondrocyte apoptosis was increased after downregulation of HDAC4. In a rat anterior cruciate ligament transection OA model, adenovirus-mediated transduction of HDAC4 was administered by intra-articular injection. We detected a stronger Safranin O staining with lower Osteoarthritis Research Society International scores, lower ATF4 and CHOP production, stronger collagen II expression, and lower chondrocyte apoptosis in rats treated with Ad-HDAC4.

**Conclusion:**

The lack of HDAC4 expression partially contributes to increased ATF4, CHOP, and endoplasmic reticulum stress-induced chondrocyte apoptosis in OA pathogenesis. HDAC4 attenuates cartilage damage by repressing ATF4-CHOP signaling-induced chondrocyte apoptosis in a rat model of OA.

**Supplementary Information:**

The online version contains supplementary material available at 10.1186/s12891-024-07578-9.

## Background

Osteoarthritis (OA) is a common degenerative articular disease characterized by the destruction of articular cartilage and osteophyte formation. Articular cartilage degeneration is the most significant pathological feature of OA [1, 2]. Studies have demonstrated that chondrocyte apoptosis plays an important role in articular cartilage degeneration. Chondrocyte apoptosis disrupts cartilage homeostasis, promoting chondrocyte apoptosis and accelerating OA progression [3–5]. However, the molecular mechanisms underlying apoptosis in chondrocytes are not completely understood.

Endoplasmic reticulum (ER) stress is defined as an imbalance between the load of unfolded proteins in the ER and the capacity of organelles to accumulate unfolded or misfolded proteins [6]. Studies have demonstrated that ER stress (ERS)-induced chondrocyte apoptosis increases as degeneration progresses in human OA cartilage [7]. Excessive and sustained ERS results in apoptosis [8–10]. Activating transcription factor 4 (ATF4) is critical in ERS-induced apoptosis [11]. Excessive or prolonged ATF4 expression upregulates the expression of its downstream target C/EBP homologous protein (CHOP), upregulating the expression of death effectors downstream of CHOP and triggering apoptosis [12–14]. However, the role of ATF4 in chondrocyte apoptosis in OA and the regulation of its expression remain unclear.

Histone deacetylase 4 (HDAC4) is a central regulator of endochondral bone formation and plays a vital role in the pathogenesis of OA [15]. A previous study has demonstrated that decreased HDAC4 expression in articular cartilage is associated with OA cartilage degeneration [16]. HDAC4 plays an important role in protecting cells against ERS-induced apoptosis. A study has demonstrated that HDAC4 interacts directly with ATF4 to prevent ATF4 translocation from the cytoplasm to the nucleus and upregulates CHOP expression. Moreover, HDAC4 inhibits ATF4 transcriptional activity [17]. Downregulation of HDAC4 increases ATF4 expression under ERS conditions and is associated with increased pro-apoptotic CHOP expression and enhanced apoptosis in multiple myeloma cells [18].

Thus, we hypothesized that lacking HDAC4 increases ERS-induced chondrocyte apoptosis by releasing ATF4 during human OA cartilage degeneration.

## Materials and methods

### Human OA cartilage specimens

The articular cartilage of the tibial plateau was obtained from patients with OA during total knee replacement. Five patients were aged 56–70 years, with an average age of 65.2 years (female). Patients with rheumatoid arthritis, hemophilia arthritis, joint tuberculosis, or infectious arthritis were excluded. Cartilage specimens extracted from severely damaged regions were divided into degraded cartilage (DC), and cartilage extracted from a relatively smooth region was divided into preserved cartilage (PC) [19]. This study was approved by the Ethics Committee of the Second Hospital of Shanxi Medical University, and each donor provided informed consent (approval no.2019YX260).

### Rat grouping experiments

An HDAC4-GFP adenoviral vector (Ad-HDAC4-GFP) was constructed and purified using GeneChem (GCPA0154819; Shanghai, China). Ad-GFP was used as the negative control adenoviral vector. Eight-week-old male Sprague-Dawley rats (*n* = 15) were purchased from the Shanxi Medical University Experimental Animal Department. All rats were housed in a controlled temperature and humidity environment with a 12-h light-dark cycle. All rats were acclimatized for one week before anterior cruciate ligament transection (ACLT). During ACLT, the rats were anesthetized with 0.3% pentobarbital sodium (1 mL/100 g) via intraperitoneal injection. ACLT and sham operations were performed on the right rat knees as described previously [20]. The rats were randomly divided into three groups (*n* = 5/group): (1) ACLT + Ad-GFP; (2) ACLT + Ad-HDAC4-GFP; and (3) sham + Ad-GFP. Ad-GFP and Ad-HDAC4-GFP were intra-articularly injected 48 h after the ACLT operation and every three weeks thereafter (1 × 10^9^ plaque-forming units/knee). All rats were euthanized by intraperitoneal injection of an overdose of pentobarbital sodium eight weeks after the operation. The verification of adenovirus-mediated transduction of HDAC4 into articular cartilage of rat knees is addressed in supplementary Materials and methods. All methods were performed in accordance with ARRIVE guidelines.

### Histology

Human cartilage samples and rat right tibial plateaus were harvested and fixed in 10% formalin for 48 h. The samples were then immersed in a 10% ethylenediaminetetraacetic acid solution for decalcification for six weeks. The rat tibial plateaus were cut into two approximately equal halves, anterior and posterior, along the frontal plane. Half of the rat tibial plateau and the human cartilage were embedded in paraffin. Then, 6-µm frontal sections were cut at 0,- 200-, 400-µm intervals for rat tibial plateaus, and serial 6-µm sections were cut for human cartilage samples. Safranin O/Fast Green staining was performed, and cartilage lesions were quantified using the Osteoarthritis Research Society International (OARSI) grading system [21].

### Immunohistochemistry (IHC)

Slides were deparaffinized with xylene and rehydrated with ethanol at different concentrations. Endogenous peroxidase was inactivated with 3% hydrogen peroxide for 10 min and then digested with 0.1% trypsin for 30 min at 37 °C for antigen retrieval. Then, 5% bovine serum albumin blocking buffer was used to block nonspecific protein binding, and the sections were then incubated with a primary antibody against HDAC4 (17449-1-AP, Proteintech), ATF4 (10835-1-AP, Proteintech), CHOP (15204-1-AP, Proteintech), or type II collagen (ab34712; Abcam) at 4 °C overnight. Thereafter, the sections were treated with horseradish peroxidase-conjugated secondary antibody for 30 min at 37 °C and developed using the 3,3′-diaminobenzidine chromogen. Images were captured using an automatic digital slide scanner (Pannoramic MIDI; 3DHISTECH, Hungary).

### Terminal deoxynucleotidyl transferase dUTP nick end labeling (TUNEL) staining

Chondrocyte apoptosis was assessed using a TUNEL apoptosis detection kit (MK1011; Boster, Wuhan, China) according to the manufacturer’s protocol. Chondrocytes with brown-yellow stained nuclei were considered apoptotic cells. Five independent human cartilage samples were used for TUNEL staining quantification.Three cartialge sections of each sample were selected. Five fields of view were selected randomly from each section. The number of apoptotic cells and total number of cells were counted, and the apoptosis index was calculated.

### Rat chondrocyte isolation and culture

Costal chondrocytes were isolated from the thorax of newborn Sprague-Dawley rats, as previously described [22]. Briefly, the ribs were dissected from the rat thorax, predigested with 0.2% collagenase II for 1 h, and further digested with 0.05% collagenase II for 3 h at 37 °C. Costal chondrocytes were cultured in DMEM/F-12 medium (Hyclone) supplemented with 10% fetal bovine serum (Gibco).

### Chondrocyte transfection

Chondrocytes at passage two were transfected with HDAC4 or HDAC4 control vector or HDAC4 siRNA or siRNA control using Lipofectamine® 3000 reagent. Briefly, chondrocytes were seeded at 1.2 × 10^5^/well in 6-well plates. At 90% confluence, the HDAC4 or HDAC4 control vector (2500 ng) or HDAC4 siRNA or siRNA control (75 pmol) were mixed with 7.5 µL of Lipofectamine 3000 reagent for transfection of cells in a single well. Then, 5 µL of P3000 Reagent was used for HDAC4 Plasmid DNA or HDAC4 control vector transfection in a single well.

### In vitro ERS model

At passage two, the chondrocytes were seeded in 6-well plates and stimulated with 300 µM H_2_O_2_ for 12 h to induce ERS. Chondrocytes in phosphate-buffered saline were used as controls. To investigate how decreased HDAC4 expression in chondrocytes affects the expression of ATF4 and chondrocyte apoptosis under ERS conditions, chondrocytes were transfected with HDAC4 or HDAC4 siRNA for 24 h. Next, the cells were stimulated with 300 µM H_2_O_2_ for 12 h. After H_2_O_2_ stimulation, cells were harvested for flow cytometry, real-time quantitative PCR (RT-qPCR), and western blotting.

### Chondrocyte apoptosis assay using flow cytometry

Chondrocyte apoptosis was measured using a Cell Apoptosis Assay Kit (KGA1104, KeyGEN, China) according to the manufacturer’s protocol. Briefly, the chondrocytes were digested using 0.25% trypsin and resuspended at a density of 1–5 × 10^6^/mL. Then, 100 µL of cell suspension was added with 5 µL of Annexin V/PE for 5 min, followed by 10 µL of 7-amino-actinomycin for 15 min. Apoptosis was detected using flow cytometry. The experiment was repeated thrice.The apoptosis rate was the sum of the % population of early-phase (Q4 quadrant: annexin V + PI−) and late-phase (Q2 quadrant: annexin V + PI+) apoptotic cells.

### RT-qPCR

Total RNA was isolated from chondrocytes and human cartilage samples using the TRIzol™ Reagent, followed by reverse transcription to complementary DNA with PrimeScript™ RT Master Mix kit (Takara, Shiga, Japan). RT-qPCR was performed using TB Green™ PCR Kit (Takara) with a two-step Real-Time PCR System (Applied Biosystems™ QuantStudio™ 6 Flex, USA). Relative transcript levels were calculated using the 2^−ΔΔCt^ method, as previously described [23]. The primer sequences are listed in Table 1.

**Table 1:** Sequences of primers

Gene	Sequence (5’-3’)
Human *HDAC4*	F: GCCAAAGATGACTTCCCTCTTA R: TTTCGGCCACTTTCTGCTTTAG
Human *ATF4*	F: ATGGATTTGAAGGAGTTCGACT R: AGAGATCACAAGTGTCATCCAA
Human *CHOP*	F: GAGAATGAAAGGAAAGTGGCAC R: ATTCACCATTCGGTCAATCAGA
Human *Caspase12*	F: AACAACCGTAACTGCCAGAGT R: CTGCACCGGCTTTTCCACT
Human *Caspase3*	F: TGTGGCATTGAGACAGAC R: CACTTGCCATACAAACTA
Human *18 S rRNA*	F: GGAGTATGGTTGCAAAGCTGA R: ATCTGTCAATCCTGTCCGTGT
Rat *GRP78*	F: CGGAGGAGGAGGACAAGAAGGAG R: ATACGACGGTGTGATGCGGTTG
Rat *ATF4*	F: GACCGAGATGAGCTTCCTGAACAG R: CCGCCTTGTCGCTGGAGAAC
Rat *CHOP*	F: CCTCGCTCTCCAGATTCCAGTCAG R: TCTCCTGCTCCTTCTCCTTCATGC
Rat *Caspase12*	F: TAGGGGAAAGTGCGAGTTTCA R: GGGCCAATCCAGCATTTACCT
Rat *HDAC4*	F: GGCTTCCTTGTGGTGGTGTTGG R: TGTACTCTCCTCGGCATGGTGTC
Rat *18 S rRNA*	F: GATGCGGCGGCGTTATTCCC R: GTGGTGCCCTTCCGTCAATTCC

### Western blotting

Total protein was isolated from the chondrocytes and quantified using a BAC Protein Assay Kit (23227, Thermo). Then, 20 µg of total protein was electrophoresed using a 10% sodium dodecyl-sulfate-polyacrylamide gel electrophoresis gel and transferred onto a polyvinylidene fluoride membrane. Nonspecific binding was blocked in Tris-buffered saline containing 0.1% Tween-20 and 5% non-fat milk at room temperature for 2 h, and then the polyvinylidene fluoride membranes were incubated at 4 °C overnight with primary antibodies against ATF4 (10835-1-AP, Proteintech), CHOP (15204-1-AP, Proteintech), and caspase-12 (bs-1105R, Bioss, China). Glyceraldehyde 3-phosphate dehydrogenase (AP0063, Bioworld, China) was used as a loading control. After washing with Tris-buffered saline containing 0.1% Tween-20, the immobilized primary antibodies were incubated with a horseradish peroxidase-conjugated secondary IgG antibody (ab205718, Abcam) at room temperature for 1 h. Immunoreactive proteins were detected using an ECL kit, and the densities of the bands were analyzed quantitatively using Image Lab software. The experiment was repeated thrice. The blots were cut prior to hybridisation with antibodies during blotting.

### Statistical analysis

An independent sample t-test was used to examine the differences in the apoptosis rate and the expression of HDAC4, GRP78, ATF4, CHOP, caspase 12 and caspase3 between the two groups. One-way analysis of variance was used to analyze the differences in OARSI scores and apoptosis rates among the three groups. The least significant difference multiple comparison test was used for pairwise comparisons following a one-way analysis of variance. Differences were considered statistically significant at *P* < 0.05. Statistical analyses were performed using SPSS version 19.0.

## Results

### Increased apoptosis of chondrocytes was found in human DC

**Fig. 1:**
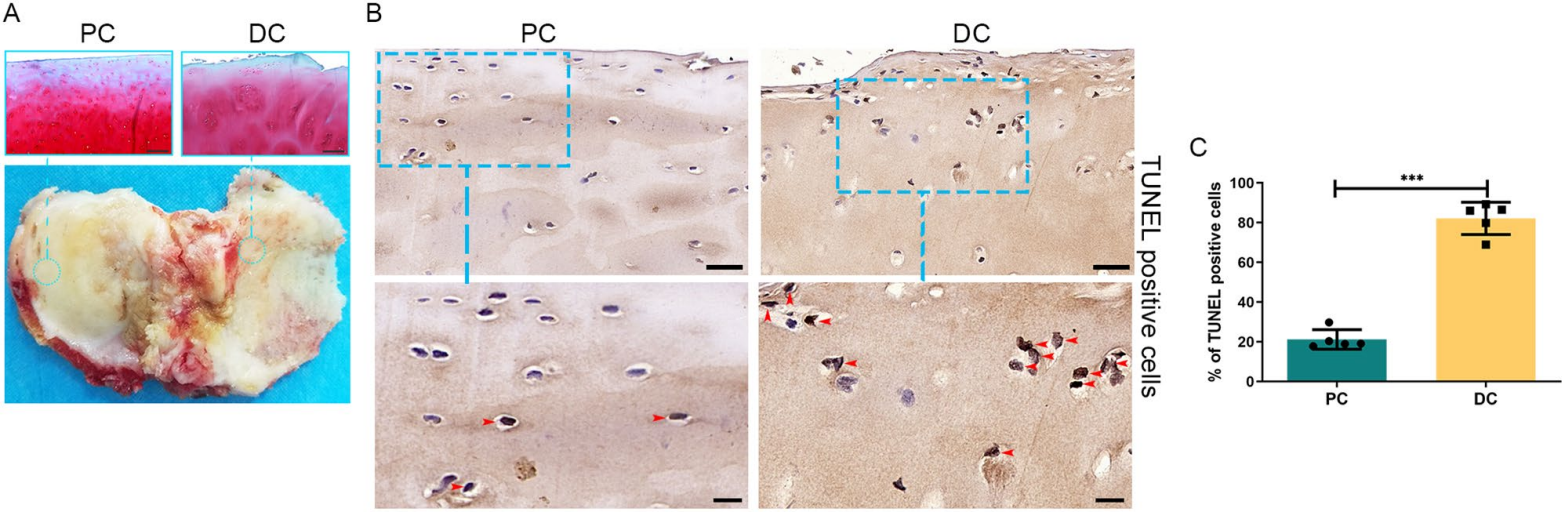
Safranin O/Fast Green staining and TUNEL staining of human articular cartilage. (**A**) Weak Safranin O staining, damaged articular cartilage surface in degraded cartilage (DC), and lack thereof in preserved cartilage (PC). The bottom panel is representative of human tibial plateau cartilage. Scale bar: 200 μm. (**B**) The TUNEL-positive staining was greater in DC than in PC (red arrow heads).The bottom panels are higher-magnification views of the boxed areas in the top panels. The top image scale bar: 50 μm; the bottom image scale bar: 20 μm. (**C**) The apoptosis rate was higher in DC than in PC. ***P < 0.001

We examined the changes in OA in the human articular cartilage using Safranin O staining. The results showed weak Safranin O staining and damaged articular cartilage surfaces in degraded cartilage and a lack thereof in PC (Fig. 1A). TUNEL staining showed that the apoptosis rate of chondrocytes was increased in DC (Fig. 1B). The apoptosis rate was higher in DC (82.15 ± 8.13%) than in PC (21.21 ± 4.90%, *P* < 0.001) (Fig. 1C).

### Increased expression of ATF4 was associated with decreased HDAC4 in human DC

**Fig. 2:**
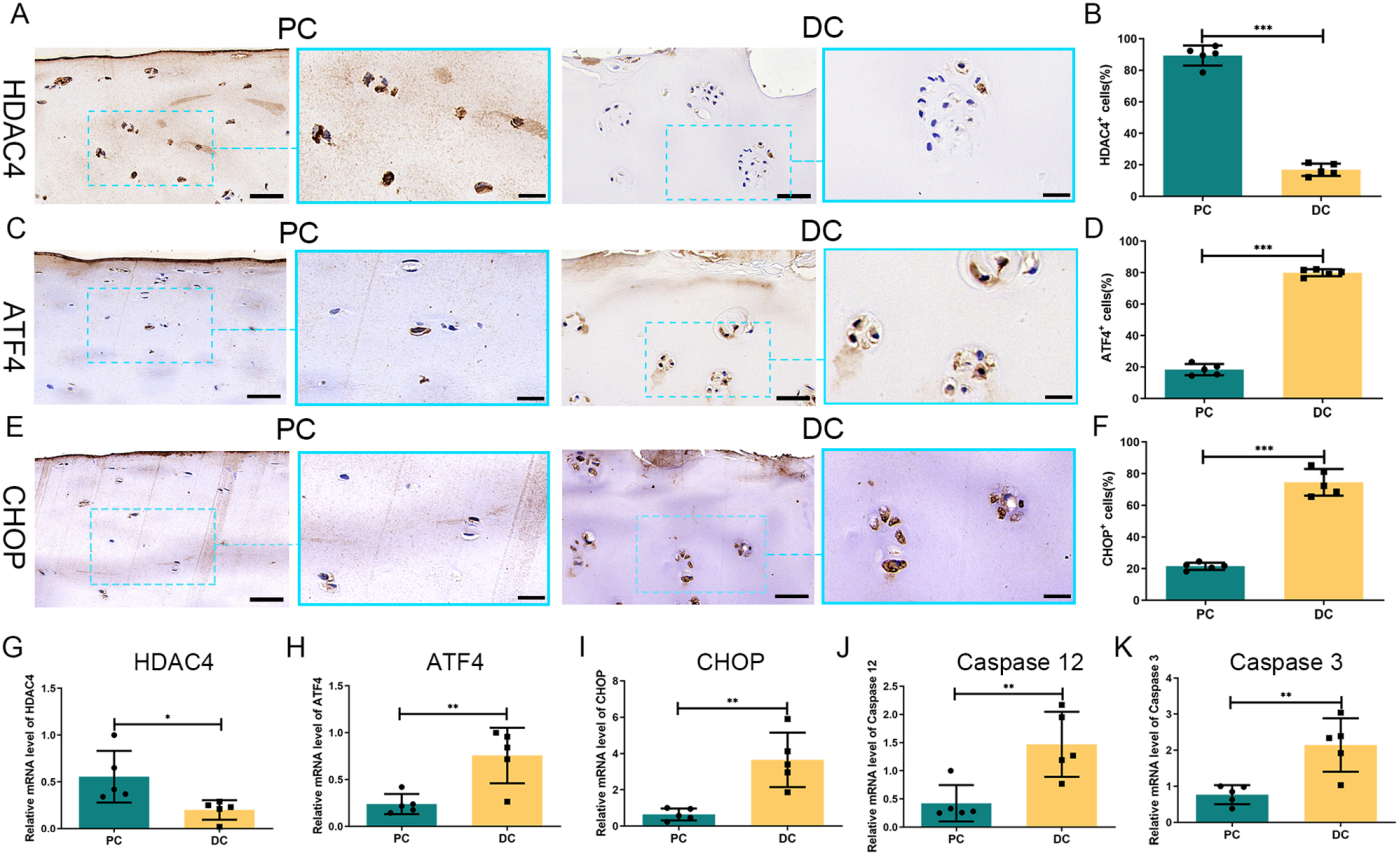
IHC staining and RT-qPCR for the expression of HDAC4, ATF4, CHOP, caspase 12, and caspase 3. (A, B) HDAC4 staining was lower in degraded cartilage (DC) than in preserved cartilage (PC). (C, D) ATF4 staining was greater in DC than in PC. (E, F) Compared with the PC, the CHOP staining was increased in DC. The right panels are higher-magnification views of the boxed areas in the left panels. The left image scale bar: 50 μm; the right image scale bar: 20 μm. (G) Gene expression of HDAC4 was decreased in DC compared to PC. (H, I, J, K) ATF4, CHOP, caspase 12, and caspase 3 mRNA levels were increased in DC compared with PC. *P < 0.05, **P < 0.01, ***P < 0.001

We used IHC and RT-qPCR to determine the expression of HDAC4 and ATF4 in the human cartilage. IHC results showed that the expression of HDAC4 was lower in DC (16.88 ± 3.92%) than in PC (89.31 ± 6.36%, *P* < 0.001) (Fig. 2A, B). In contrast, ATF4 expression was higher in DC (80.00 ± 2.24%) than in PC (18.37 ± 3.58%, *P* < 0.001) (Fig. 2C, D). The RT-qPCR yielded similar results. The gene expression of HDAC4 decreased by 64.30% in DC compared with that in PC (Fig. 2G). ATF4 gene expression increased 3.16-fold in DC compared with PC (Fig. 2H).

### The expression levels of CHOP, caspase 12, and caspase 3 were increased in human OA cartilage

IHC staining showed that CHOP expression was significantly higher in the DC (74.56 ± 8.41%) than in the PC (21.52 ± 2.28%, *P* < 0.001) (Fig. 2E, F). RT-qPCR further confirmed the IHC results, which showed that CHOP expression was higher in the DC than in the PC (*P* < 0.01) (Fig. 2I). The gene expression levels of caspase12 and caspase 3 were higher in the DC than in the PC(*P* < 0.01) (Fig. 2J, K).

### RT-qPCR indicates that H_2_O_2_ induces in vitro ERS

RT-qPCR results showed that the gene expression levels of GRP78, ATF4, CHOP, and caspase 12 were higher in H_2_O_2_-treated chondrocytes than in the control group, indicating that H_2_O_2_ could induce ERS in vitro (Supplementary Fig. 1A, B,C, D). The transfection efficiency of HDAC4 and siRNA was determined using RT-qPCR. The results showed that after transfection with HDAC4 into chondrocytes, HDAC4 mRNA was increased 42-fold compared with the control group, while HDAC4 gene expression was reduced by 63.20% after transfection with HDAC4 siRNA (Supplementary Fig. 1E, F).

### HDAC4 represses ATF4 expression in chondrocytes under ERS

**Fig. 3:**
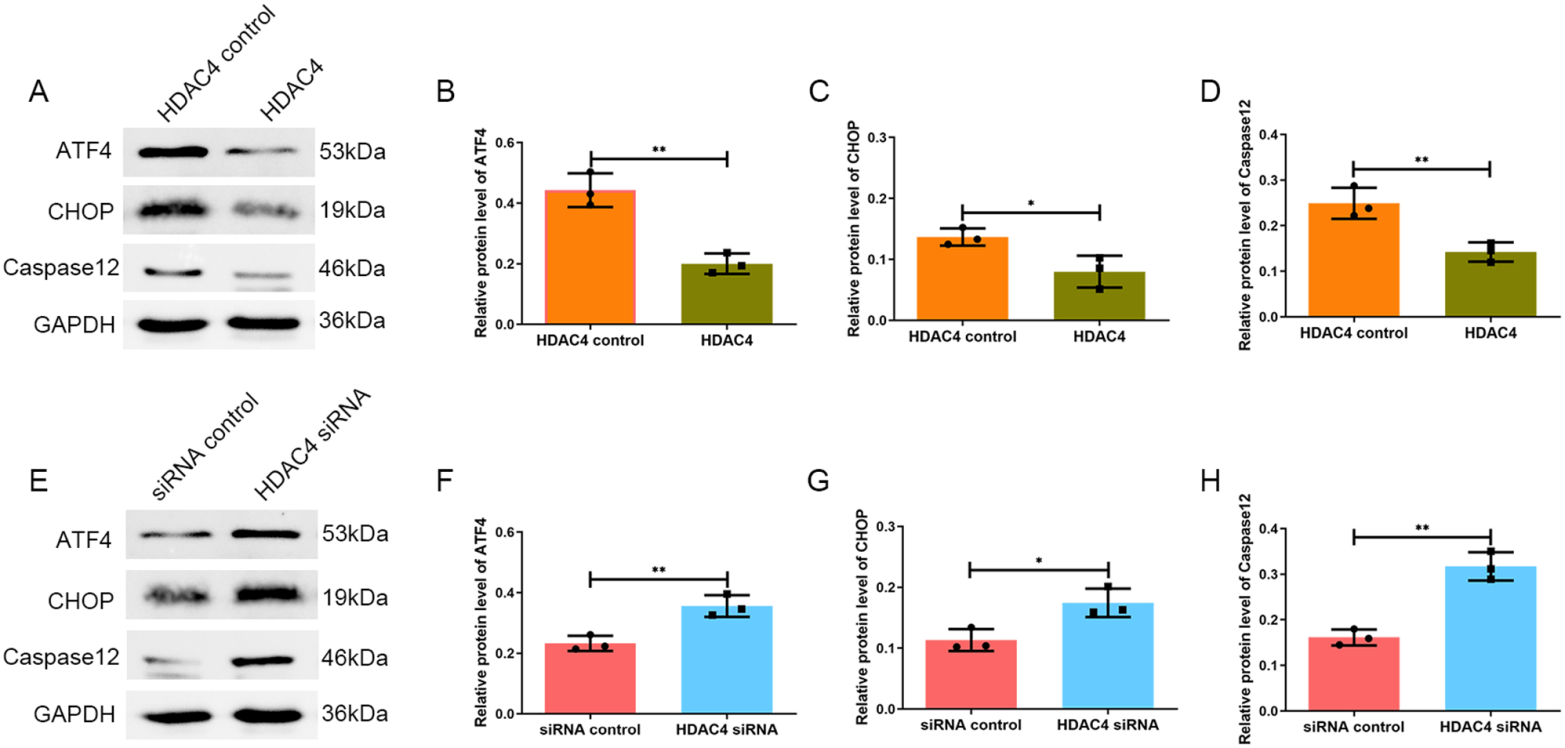
(A, B) Western blotting showed that the ATF4 expression was lower in chondrocytes transfected with HDAC4 than in the control group. (E, F) Increased protein expression of ATF4 was detected in chondrocytes transfected with HDAC4 siRNA compared with the control group. (A, C,D) Western blotting results showed that upregulation of HDAC4 decreased the expression of CHOP and caspase 12 under ERS. (E, G,H) Downregulation of HDAC4 increased the protein expression of CHOP and caspase 12.*P < 0.05, **P < 0.01

To verify whether the lack of HDAC4 expression was related to increased ATF4 expression in OA cartilage, chondrocytes were transfected with HDAC4 expression plasmid or HDAC4 siRNA to upregulate or downregulate the expression of HDAC4. Western blotting showed that the ATF4 expression was lower in chondrocytes transfected with HDAC4 than in the control group (*P* < 0.01) (Fig. 3A, B). Increased protein expression of ATF4 was detected in chondrocytes transfected with HDAC4 siRNA compared with that in the control group (*P* < 0.01) (Fig. 3E, F).

### The expression of CHOP and caspase 12 was decreased after HDAC4 upregulation in chondrocytes under in vitro ERS

We detected the expression of CHOP and caspase 12 using western blotting. The results showed that the upregulation of HDAC4 decreased the protein expression of CHOP(*P* < 0.05) and caspase 12 (*P* < 0.01) under ERS (Fig. 3A, C,D). Downregulation of HDAC4 increased the protein expression of CHOP (*P* < 0.05) and caspase 12(*P* < 0.01) (Fig. 3E, G,H).

### HDAC4 decreased the chondrocyte apoptosis rate under ERS

**Fig. 4:**
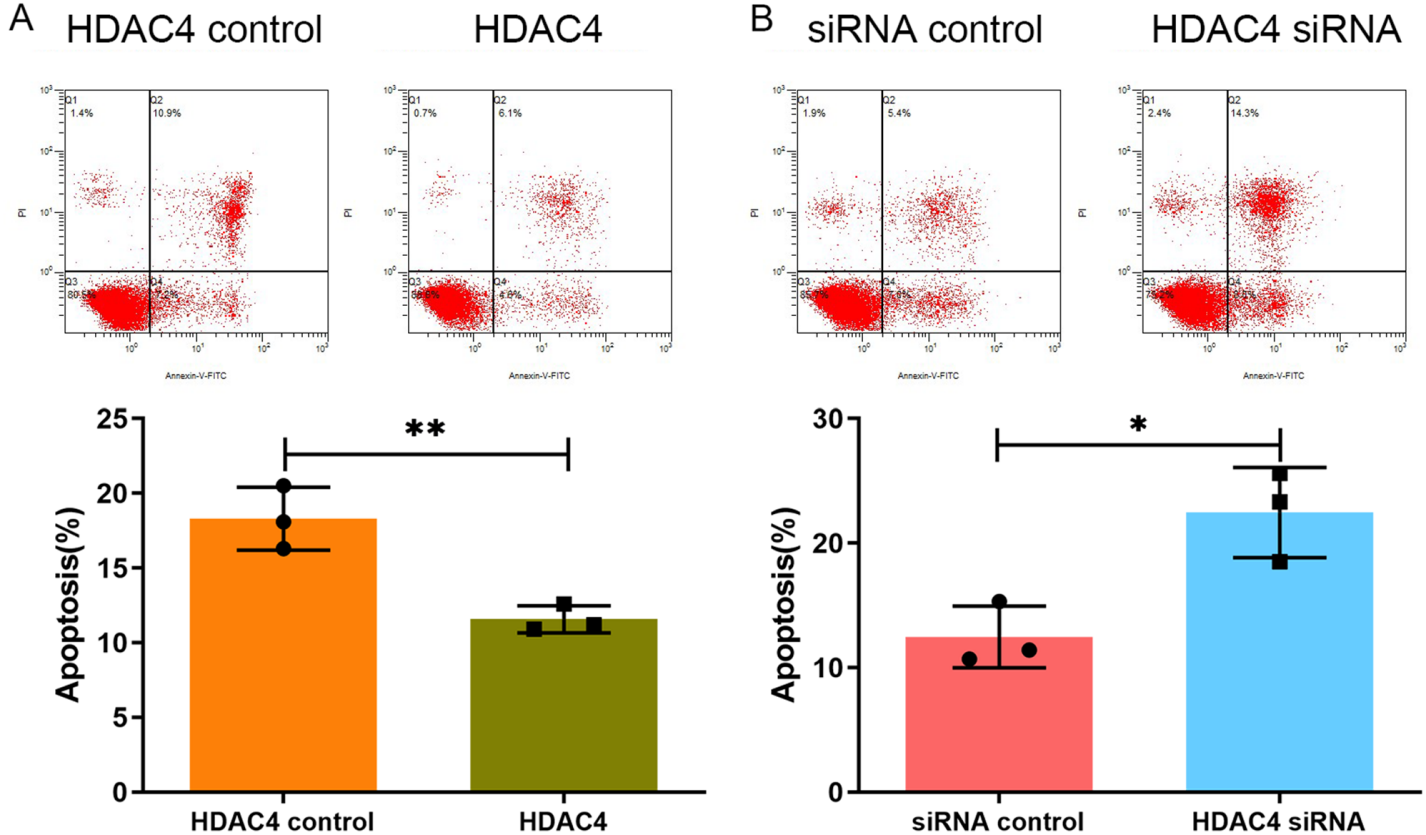
Flow cytometry was used to measure the chondrocyte apoptosis. (**A**) In HDAC4-treated groups, the chondrocyte apoptosis rate was lower than in the control group (18.30 ± 2.11% vs. 11.57 ± 0.91%). (**B**) Downregulation of HDAC4 in chondrocytes increased the apoptosis rate compared with the control group (12.47 ± 2.48% vs. 22.47 ± 3.62%).*P < 0.05,**P < 0.01

Flow cytometry was used to measure chondrocyte apoptosis. The results showed that upregulation of HDAC4 reduced chondrocyte apoptosis under ER stress. In HDAC4-treated groups, the chondrocyte apoptosis rate was lower than in the control group (18.30 ± 2.11% vs. 11.57 ± 0.91%, *P* < 0.01) (Fig. 4A). Downregulation of HDAC4 in chondrocytes increased the apoptosis rate compared with the control group (12.47 ± 2.48% vs. 22.47 ± 3.62%, *P* < 0.05) (Fig. 4B).

### HDAC4 attenuated the degeneration of articular cartilage by repressing the ATF4/CHOP pathway in a rat OA model

**Fig. 5:**
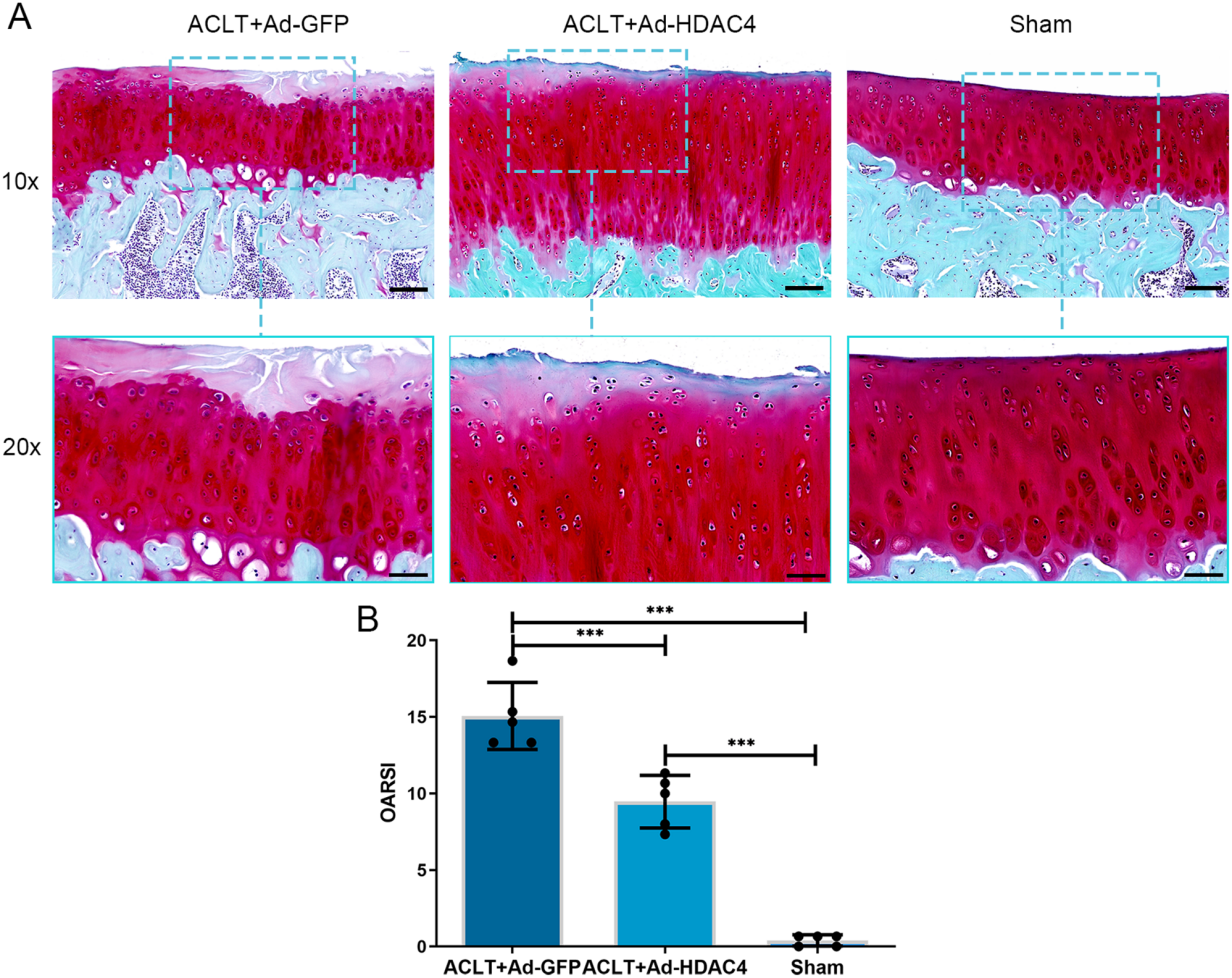
HDAC4 attenuated the degeneration of articular cartilage. (**A**) Strong Safranin O staining and more intact articular cartilage surface in the Ad-HDAC4 group compared with the Ad-GFP group. 10× scale bar: 100 μm, 20× scale bar: 50 μm. (**B**) The OARSI scores were significantly lower in the Ad-HDAC4 group than in the Ad-GFP group. Sham group had the lowest OARSI score.***P < 0.001

**Fig. 6:**
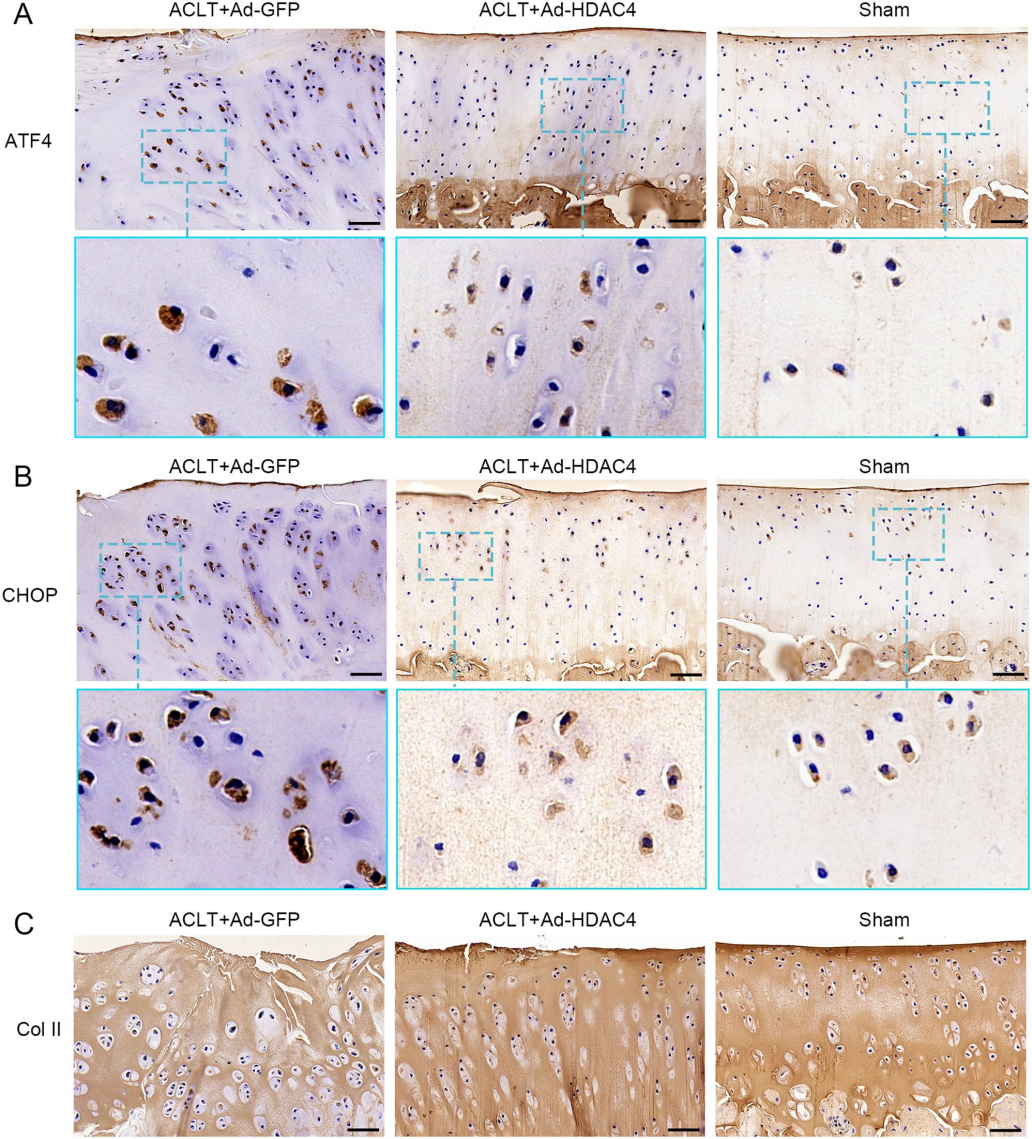
HDAC4 decreased the expression of ATF4 and CHOP and increased the expression of collagen II (Col II). (A, B) The expression of ATF4 and CHOP were lower in the Ad-HDAC4 and sham groups than in the Ad-GFP group. (C) Col II staining was greater in the Ad-HDAC4 and sham-operated groups than in the Ad-GFP group. Scale bar: 50 μm

**Fig. 7:**
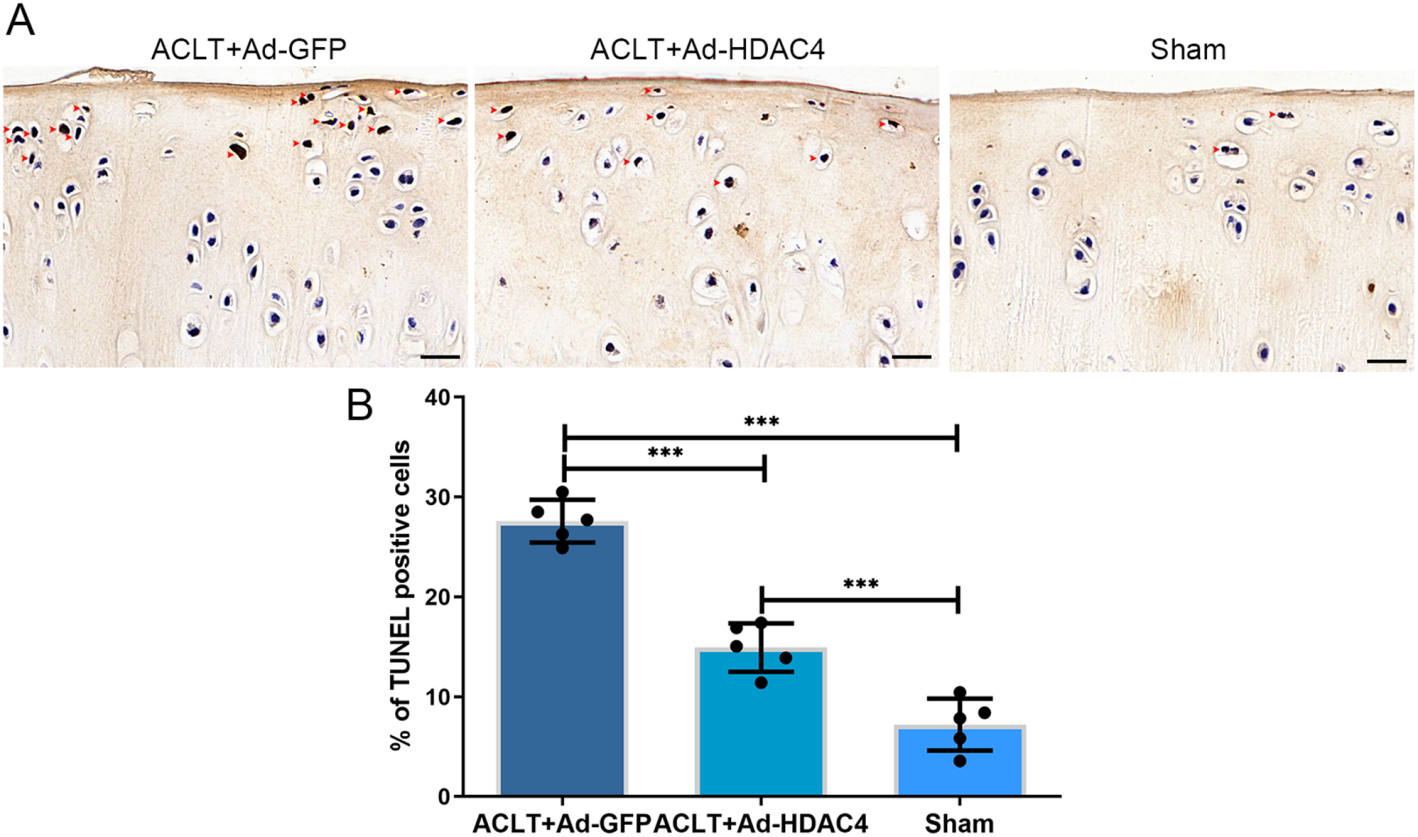
HDAC4 decreases the chondrocyte apoptosis in an ACLT rat OA model. (A) TUNEL staining results showed that the TUNEL-positive staining (red arrow heads) in the Ad-HDAC4 and Sham groups was lower than that in the Ad-GFP group. Scale bar: 20 μm. (B) The chondrocyte apoptosis rate was significantly lower in the Ad-HDAC4 and Sham groups than in the Ad-GFP group. ***P < 0.001

We further tested HDAC4 in vivo by intra-articular injection in a rat ACLT-OA model. We successfully introduced the HDAC4 gene into the rat articular cartilage, as indicated by green fluorescence at two weeks after injection (Supplementary Fig. 1G, H). Safranin O staining revealed that the articular cartilage surface was more intact in the Ad-HDAC4 group than in the Ad-GFP group (Fig. 5A). The resulting OARSI scores were significantly lower in the Ad-HDAC4 group (9.47 ± 1.73, maximum score: 11.33) than in the Ad-GFP group (15.07 ± 2.19, *P* < 0.001, maximum score: 18.67). The Sham group had the lowest OARSI score (0.40 ± 0.37; *P* < 0.001, maximum score: 0.67) (Fig. 5B). IHC staining showed that ATF4 and CHOP expression were lower in the Ad-HDAC4 and sham groups than in the Ad-GFP group (Fig. 6A, B). In contrast, collagen II staining was elevated in the Ad-HDAC4 and sham groups compared with the Ad-GFP group (Fig. 6C). We compared chondrocytes apoptosis in the three groups using TUNEL staining (Fig. 7A). The results showed that chondrocyte apoptosis was significantly lower in the Ad-HDAC4 group (14.93 ± 2.42%) and Sham group (7.21 ± 2.61%) than in the Ad-GFP group (27.57 ± 2.14%, *P* < 0.001) (Fig. 7B).

## Discussion

HDAC4 is a member of the class IIa HDAC family and is mainly expressed in the brain, muscle, and cartilage [24, 25]. Studies have demonstrated that HDAC4 plays a vital role in endochondral bone formation [15] and that decreased HDAC4 expression in articular cartilage is associated with OA pathogenesis [16]. In this study, we showed that HDAC4 expression significantly decreased in DC, which is consistent with a previous study [16]. TUNEL staining showed that the chondrocyte apoptosis rate was higher in DC than in PC. Based on these results in human cartilage samples, we speculated that decreased HDAC4 expression might be associated with increased chondrocyte apoptosis in OA cartilage. Chondrocyte apoptosis disrupts the balance between catabolic and anabolic processes in cartilage and promotes cartilage destruction [26–28].

In addition to decreased HDAC4 and increased chondrocyte apoptosis, we found that the expression of ERS-related factors, such as ATF4, CHOP, and caspase 12, was increased in DC compared with that in PC. Chondrocytes are located in a unique microenvironment without blood vessels and nerves [29]. Therefore, chondrocytes are extremely sensitive to stimulation with various physical and chemical factors, which can easily lead to ERS [30]. A previous study has demonstrated that ERS in chondrocytes increases during the progression of OA, resulting in increased chondrocyte apoptosis [31]. ATF4 is a critical regulator for ERS-induced apoptosis. When ERS is prolonged, activated protein kinase RNA-like ER kinase phosphorylates translation initiation factor 2α, which selectively facilitates the ATF4; thus, the ERS response is activated, and the apoptosis process is initiated [32–34]. ATF4 plays an essential role in the regulation of CHOP expression. CHOP downregulates B-cell lymphoma 2 expression and increases the translocation of bcl-2-like protein 4 from the cytosol to the mitochondria, which triggers the activation of caspase 3, resulting in apoptosis [13, 35, 36]. These findings indicate that increased ATF4 expression in OA cartilage may trigger the expression of a series of genes related to chondrocyte apoptosis. However, the mechanism by which ATF4 is regulated in OA cartilage is unknown. Previous studies have demonstrated that HDAC4 negatively regulates apoptosis by directly interacting with and repressing ATF4 [17]. Downregulation of HDAC4 increases ATF4 expression under ERS, which is associated with increased pro-apoptotic CHOP expression and enhanced apoptosis of multiple myeloma cells [18]. Based on these findings, it is likely that decreased HDAC4 expression is responsible for increased ATF4 expression and chondrocyte apoptosis in OA cartilage.

To test whether decreased HDAC4 expression was related to increased ATF4 and chondrocyte apoptosis in OA pathogenesis, rat chondrocytes were transfected with either an HDAC4 expression construct or siRNA against HDAC4. Our results showed that under ERS, the upregulation of HDAC4 in chondrocytes decreased the expression of ATF4, while the downregulation of HDAC4 in chondrocytes increased the expression of ATF4. The expression of CHOP, which is downstream of ATF4, exhibited a similar trend. Caspase 12 is located on the outer surface of the ER membrane and is activated by ER stressors that are specific to ERS-mediated apoptosis [37, 38]. In this study, we found that the expression of caspase 12 was increased after HDAC4 downregulation and decreased when HDAC4 was upregulated in chondrocytes. The chondrocyte apoptosis was reduced after HDAC4 upregulation; the opposite effect was observed when HDAC4 was knocked down by siRNA.

This study found that decreased HDAC4 expression contributes to increased levels of ATF4, CHOP, caspase 12, and apoptosis in OA chondrocytes. Overexpression of HDAC4 reduces the expression of ATF4, CHOP, and caspase 12 and chondrocyte apoptosis. We further tested the effect of HDAC4 on cartilage degeneration by repressing the ATF4-CHOP pathway-induced chondrocyte apoptosis in a rat model of OA. The results showed less cartilage damage and lower OARSI scores in Ad-HDAC4-treated rats. The expression of ATF4 and CHOP decreased in the articular cartilage of HDAC4-treated rats. Chondrocyte apoptosis in the rat articular cartilage was detected using TUNEL staining. Reduced chondrocyte apoptosis was found in Ad-HDAC4-treated rats compared with Ad-GFP-treated rats. In addition, the collagen II expression was upregulated in Ad-HDAC4-treated rats. These results indicated that HDAC4 inhibited cartilage degeneration by blocking ATF4-CHOP signaling-induced chondrocyte apoptosis.

This study has some limitations. First, ATF4-CHOP signaling-induced chondrocyte apoptosis was not completely suppressed by HDAC4. The ERS-induced apoptosis is also induced by c-Jun N-terminal kinases other than CHOP [39]. In-depth studies are required to clarify the role of ERS-mediated apoptosis in the pathogenesis of OA. Second, the ACLT-OA model is a traumatic joint instability model that cannot fully simulate the degenerative pathological processes of human OA. This may affect the chondroprotective effects of HDAC4 in OA therapy.

## Conclusion

In conclusion, our findings indicate that the lack of HDAC4 expression partially contributes to increased ATF4, CHOP, and ERS-induced chondrocyte apoptosis in human OA cartilage. HDAC4 attenuates articular cartilage damage by repressing ATF4-CHOP signaling-induced chondrocyte apoptosis in a rat model of OA. The upregulation of HDAC4 may have chondroprotective effects in patients with OA.

### Electronic supplementary material

Below is the link to the electronic supplementary material.

Supplementary Material 1

Supplementary Material 2

## Data Availability

The datasets used and/or analysed during the current study are available from the corresponding author on reasonable request.

## Abbreviations

OA: Osteoarthritis
HDAC4: Histone deacetylase 4
ATF4: Activating transcription factor 4
CHOP: C/EBP homologous protein
ERS: Endoplasmic reticulum stress
Col II: Type II collagen
ACLT: Anterior cruciate ligament transection
