# Supplementary figures and images for: HDAC4 represses ER stress induced chondrocyte apoptosis by inhibiting ATF4 and attenuates cartilage degeneration in an osteoarthritis rat model
Source: BMC Musculoskelet Disord. 2024 Jun 15;25:467. doi: 10.1186/s12891-024-07578-9 (PMC11179397; doi:10.1186/s12891-024-07578-9)

**WB original images**

**GAPDH**


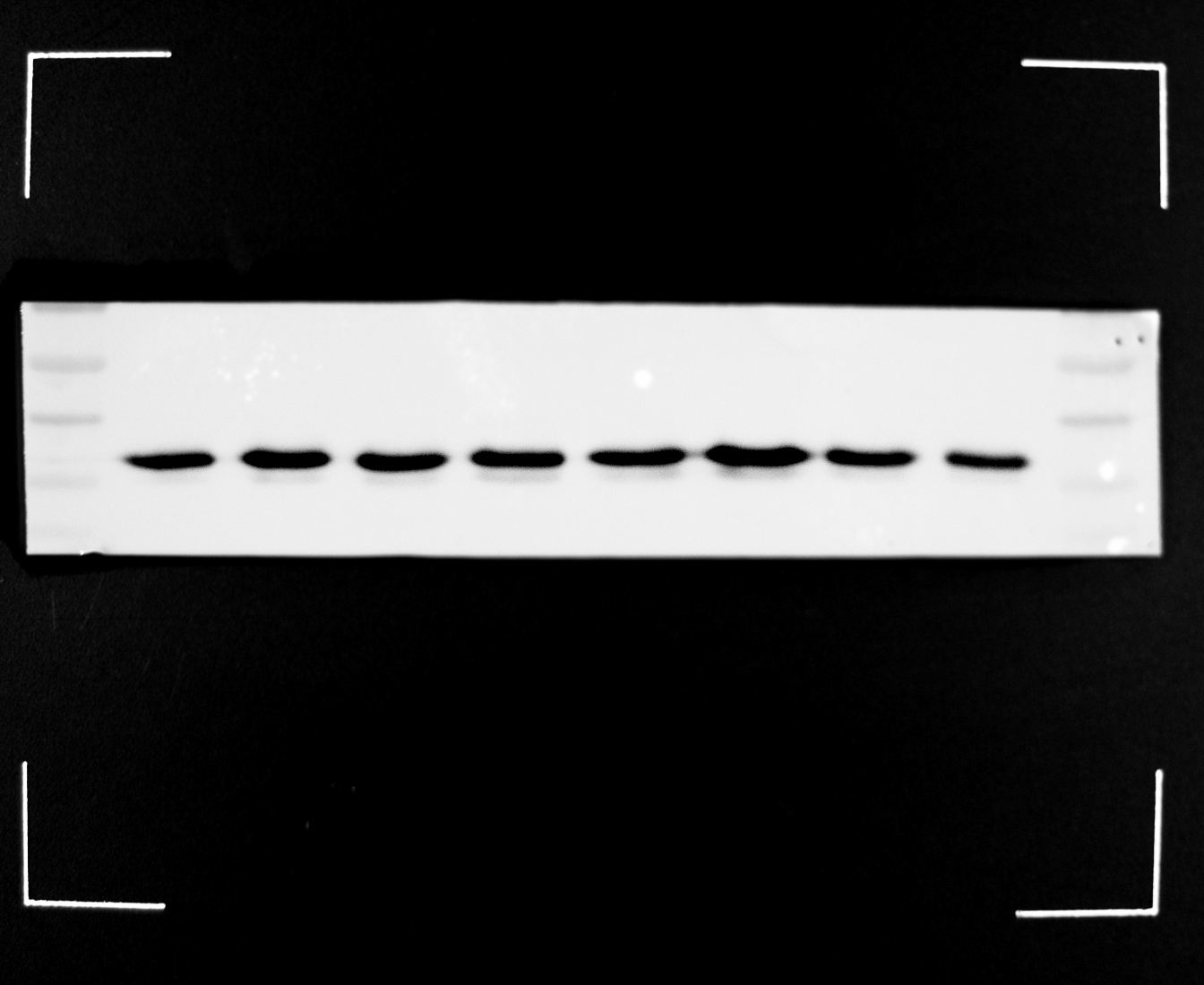
 **
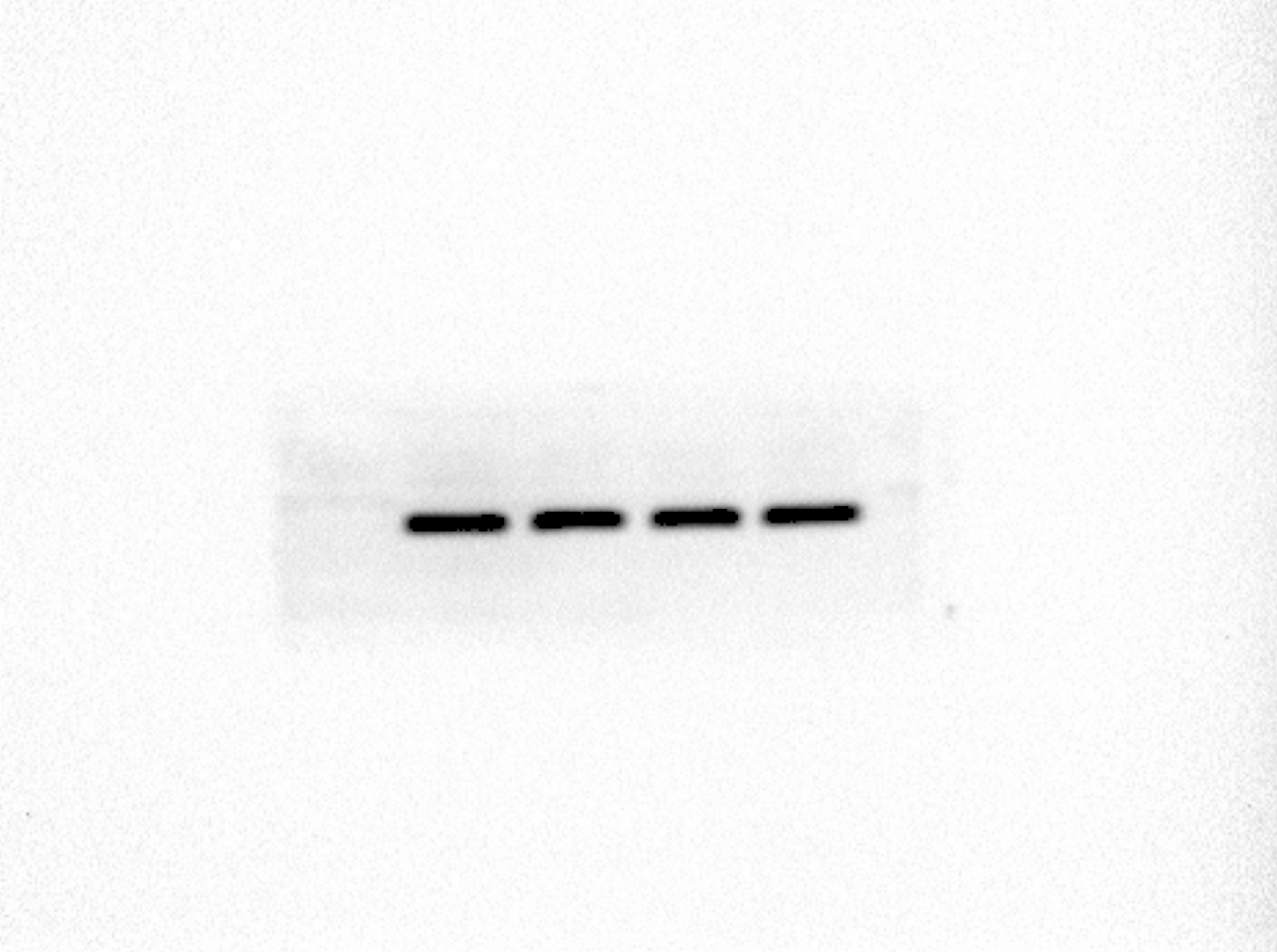
**

36kDa

36kDa

**ATF4**


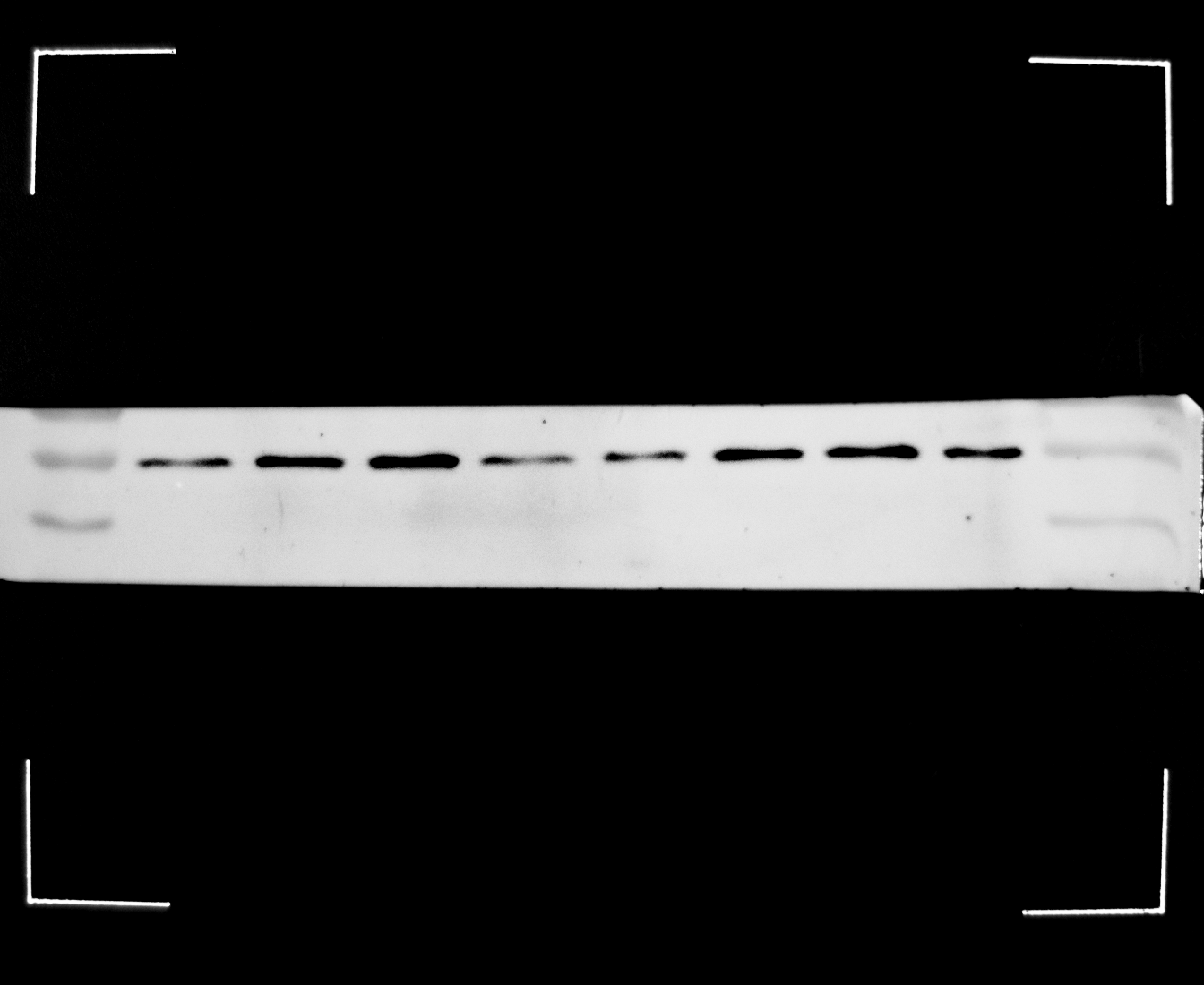
 **
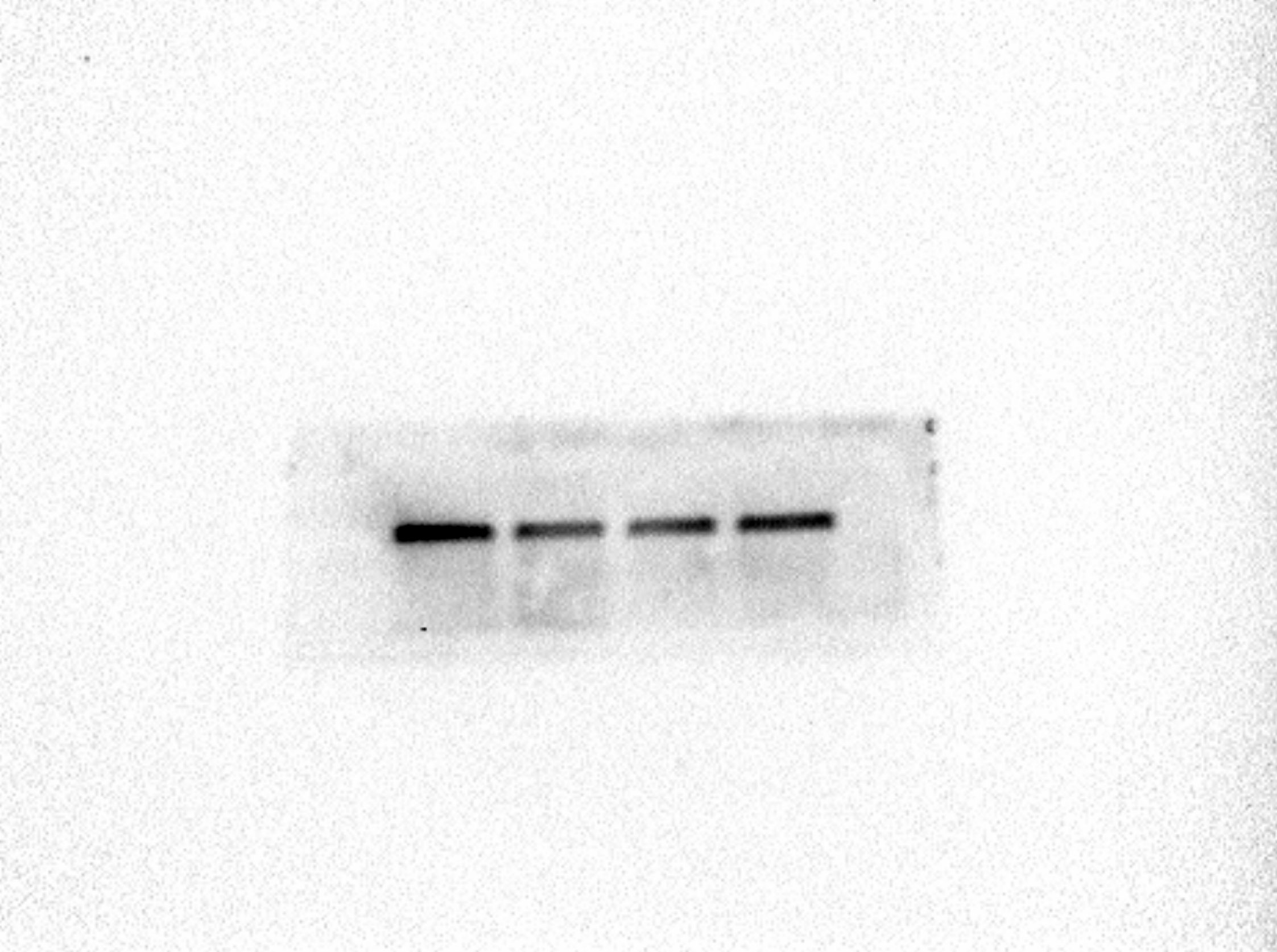
**

53kDa

53kDa

**CHOP**


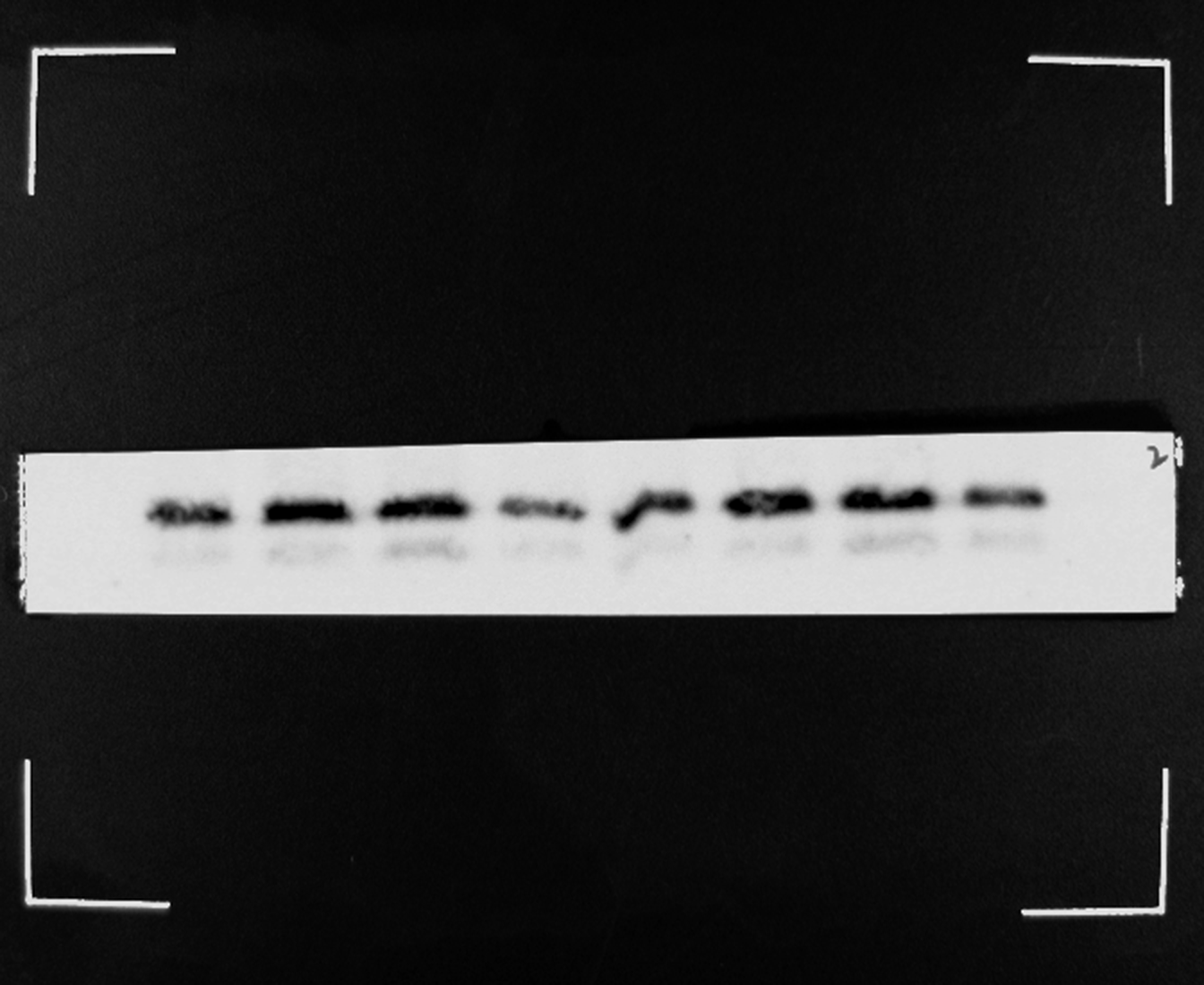
 **
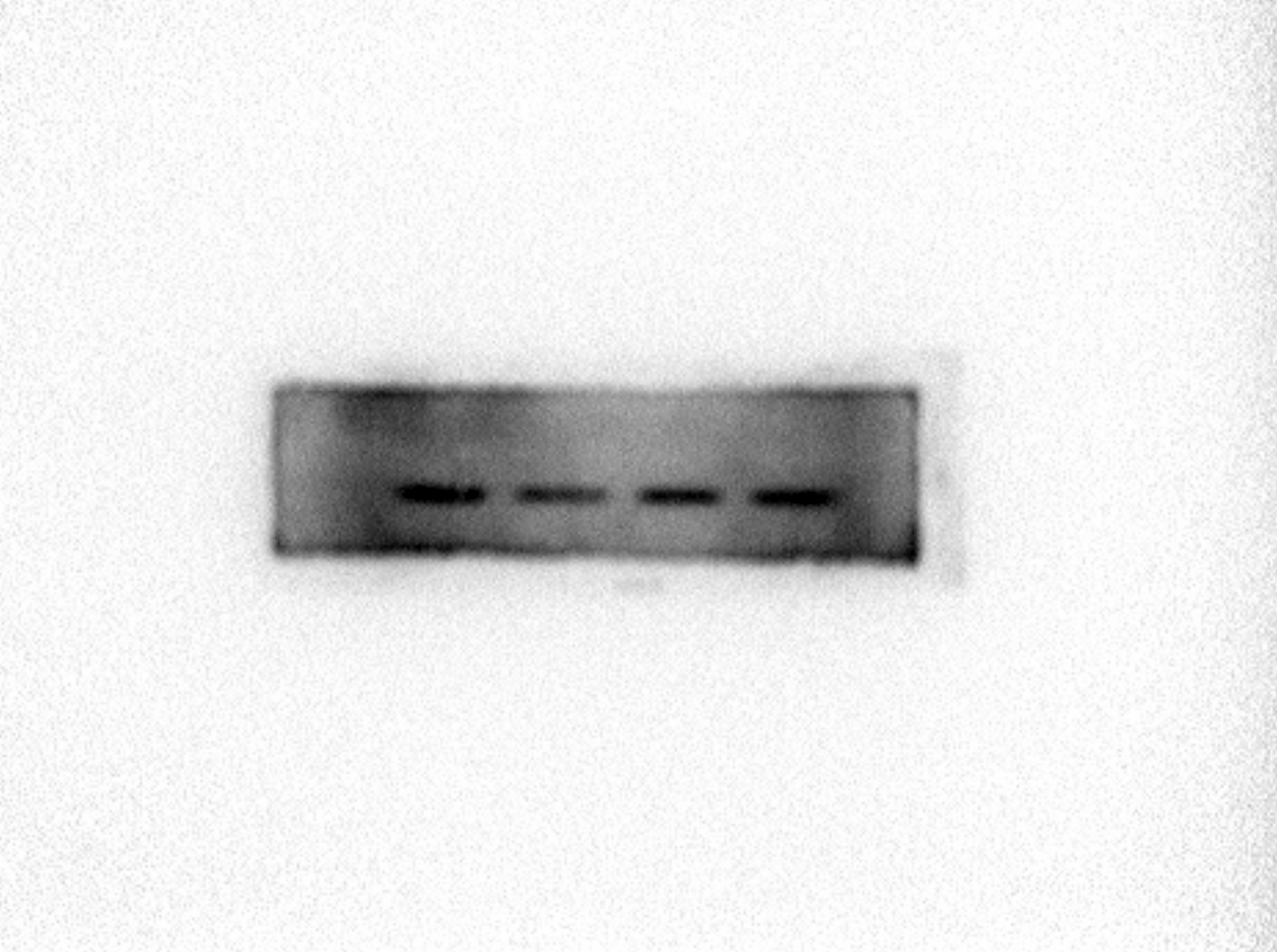
**

19kDa

19kDa

**Caspase12**


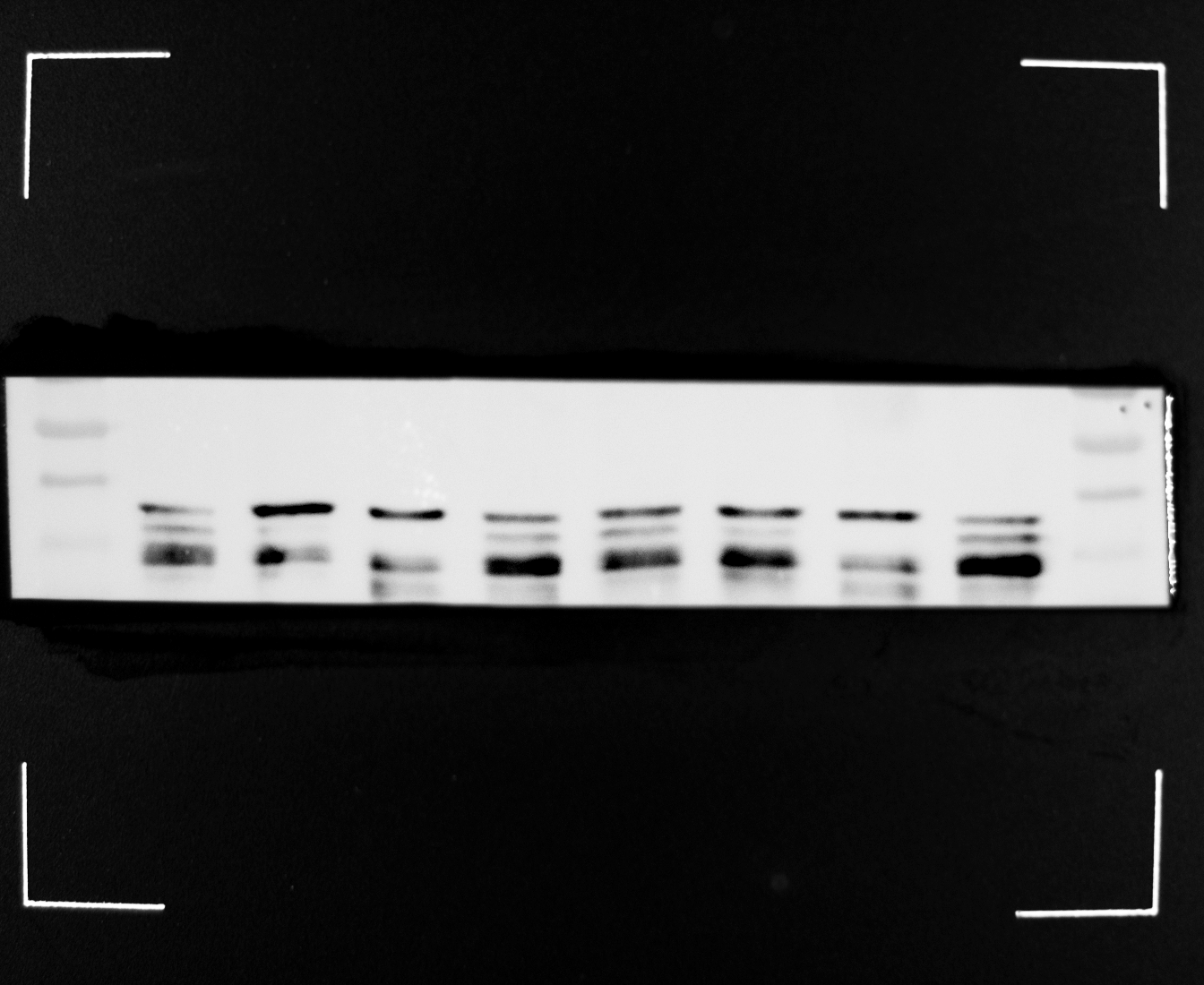
 **
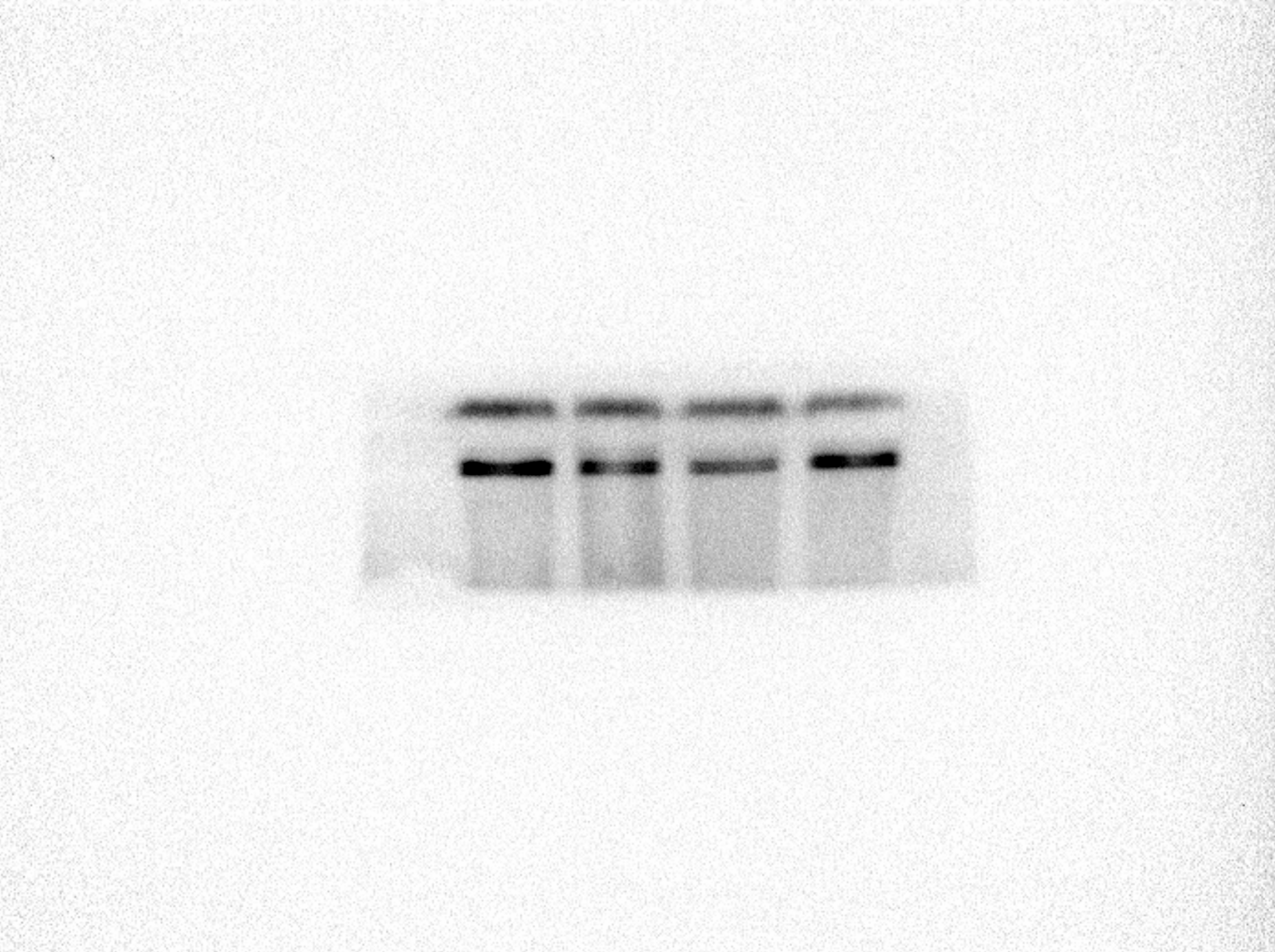
**

46kDa

46kDa

Supplement: Supplementary file 1 — Supplementary Material 1 [file 12891_2024_7578_MOESM1_ESM.doc]
