# Supplementary material for: HDAC4 represses ER stress induced chondrocyte apoptosis by inhibiting ATF4 and attenuates cartilage degeneration in an osteoarthritis rat model
Source: BMC Musculoskelet Disord. 2024 Jun 15;25:467. doi: 10.1186/s12891-024-07578-9 (PMC11179397; doi:10.1186/s12891-024-07578-9)
Supplement: Supplementary file 2 — Supplementary Material 2 [file 12891_2024_7578_MOESM2_ESM.docx]

**Supplementary Materials and methods**

**Adenovirus HDAC4 transduction of the articular cartilage of a rat model of OA.**

Eight-week-old male Sprague-Dawley rats were purchased from the Shanxi Medical University Experimental Animal Department. OA was induced by anterior cruciate ligament transection (ACLT) on the right rat knees. During ACLT, the rats were anesthetized with 0.3% pentobarbital sodium (1 mL/100 g) via intraperitoneal injection. The rats were randomly divided into three groups (n = 3/group): (1) ACLT+Ad-GFP; (2) ACLT+Ad-HDAC4-GFP; and (3) Sham+Ad-GFP. Ad-GFP and Ad-HDAC4-GFP were intra-articularly injected 48 h after the ACLT operation (1×10^9^ plaque-forming units/knee). All rats were sacrificed by intraperitoneal injection of an overdose of pentobarbital sodium two weeks after the adenovirus injection. The tibial plateaus were harvested for histological examination of 6-μm sections to determine the location and expression of HDAC4 in the rat knee using fluorescence microscopy. The femoral condyle cartilage was harvested, and the expression of HDAC4 was examined by RT-qPCR.

**Supplementary Figure and Legend**

**Supplementary Fig. 1**


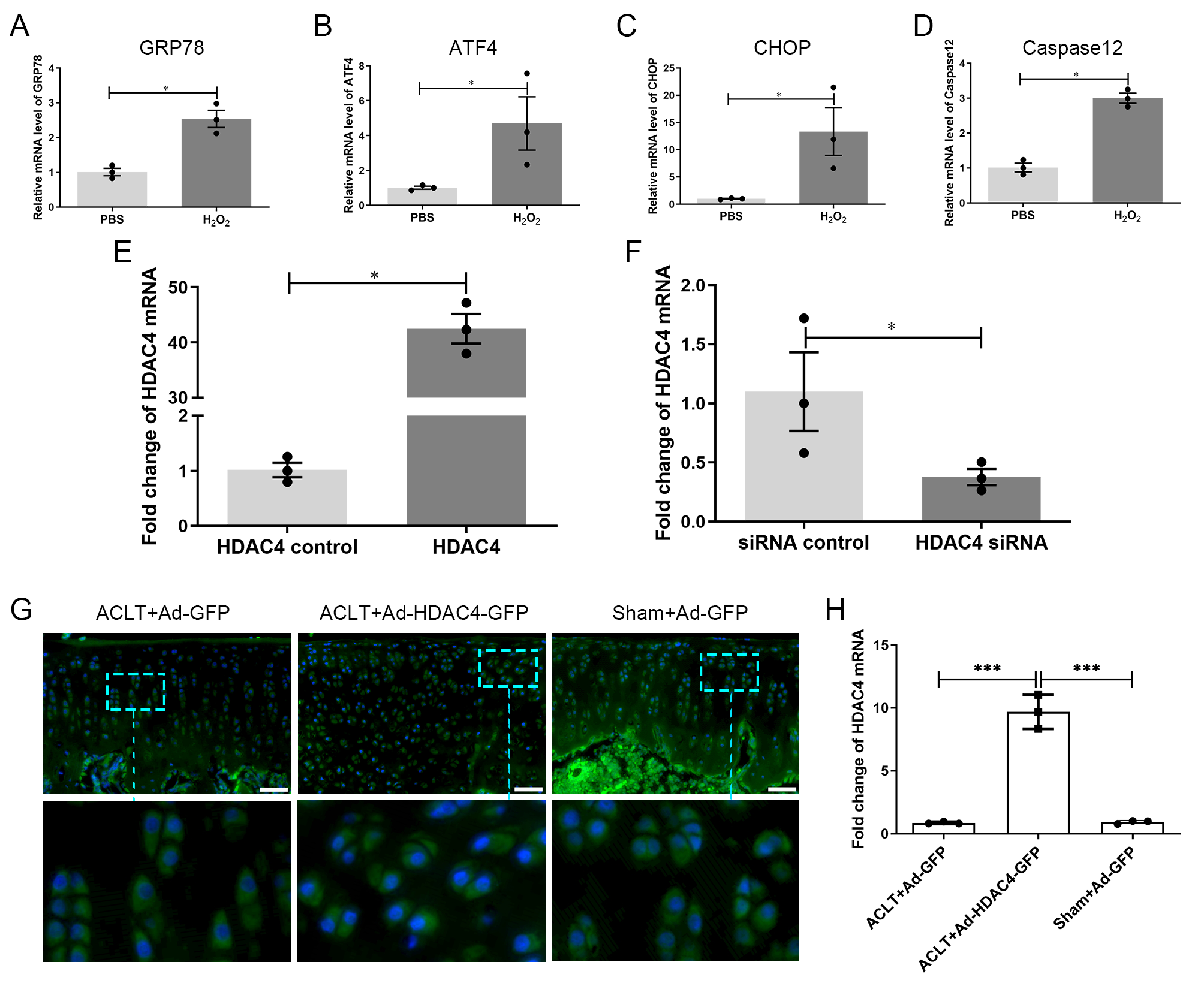


**Supplementary Fig. 1** The mRNA levels of GRP78 (A), ATF4 (B), CHOP (C), and caspase 12 (D) were increased in H_2_O_2_-treated chondrocytes, which indicated that H_2_O_2_ can induce in vitro endoplasmic reticulum stress. (E) HDAC4 mRNA was increased 42-fold compared with the control group. (F) HDAC4 gene expression was reduced by 63.20% after being transfected with HDAC4 siRNA.(G) Fluorescence microscopy shows strong GFP (green fluorescent protein) expression at 2 weeks after Ad-GFP or Ad-HDAC4-GFP injection. The bottom panels are higher-magnification view of the boxed area in the top panels. Scale bars: 50 μm. (H) At 2 weeks after the adenovirus injection, total RNA was isolated from the cartilage of the ACLT+Ad-GFP, ACLT+Ad-HDAC4-GFP and Sham+Ad-GFP groups. RT-qPCR results show the level of HDAC4 was significantly higher in the Ad-HDAC4-GFP group than in the ACLT+Ad-GFP and Sham+Ad-GFP groups. **P*<0.05, ****P*<0.001.
